# Statistical Methods for Establishing Personalized Treatment Rules in Oncology

**DOI:** 10.1155/2015/670691

**Published:** 2015-09-13

**Authors:** Junsheng Ma, Brian P. Hobbs, Francesco C. Stingo

**Affiliations:** Department of Biostatistics, The University of Texas MD Anderson Cancer Center, Unit 1411, 1400 Pressler Street, Houston, TX 77030, USA

## Abstract

The process for using statistical inference to establish personalized treatment strategies requires specific techniques for data-analysis that optimize the combination of competing therapies with candidate genetic features and characteristics of the patient and disease. A wide variety of methods have been developed. However, heretofore the usefulness of these recent advances has not been fully recognized by the oncology community, and the scope of their applications has not been summarized. In this paper, we provide an overview of statistical methods for establishing optimal treatment rules for personalized medicine and discuss specific examples in various medical contexts with oncology as an emphasis. We also point the reader to statistical software for implementation of the methods when available.

## 1. Introduction

Cancer is a set of diseases characterized by cellular alterations the complexity of which is defined at multiple levels of cellular organization [1, 2]. Personalized medicine attempts to combine a patient's genomic and clinical characteristics to devise a treatment strategy that exploits current understanding of the biological mechanisms of the disease [3, 4]. Recently the field has witnessed successful development of several molecularly targeted medicines, such as Trastuzumab, a drug developed to treat breast cancer patients with* HER2* amplification and overexpression [5, 6]. However, successes have been limited. Only 13% of cancer drugs that initiated phase I from 1993 to 2004 attained final market approval by the US Food and Drug Administration (FDA) [7]. Moreover, from 2003 to 2011, 71.7% of new agents failed in phase II, and only 10.5% were approved by the FDA [8]. The low success rate can be partially explained by inadequate drug development strategies [3] and an overreliance on univariate statistical models that fail to account for the joint effects of multiple candidate genes and environmental exposures [9]. For example, in colorectal cancer there have been numerous attempts to develop treatments that target a single mutation, yet only one, an EGFR-targeted therapy for metastatic disease, is currently used in clinical practice [10].

In oncology, biomarkers are typically classified as either predictive or prognostic. Prognostic biomarkers are correlates for the extent of disease or extent to which the disease is curable. Therefore, prognostic biomarkers impact the likelihood of achieving a therapeutic response regardless of the type of treatment. By way of contrast, predictive biomarkers select patients who are likely or unlikely to benefit from a particular class of therapies [3]. Thus, predictive biomarkers are used to guide treatment selection for individualized therapy based on the specific attributes of a patient's disease. For example, BRAF V600-mutant is a widely known predictive biomarker which is used to guide the selection of Vemurafenib for treatment metastatic melanoma [11]. Biomarkers need not derive from single genes as those aforementioned and yet may arise from the combination of a small set of genes or molecular subtypes obtained from global gene expression profiles [6]. Recently, studies have shown that the Oncotype DX recurrence score, which is based on 21 genes, can predict a woman's therapeutic response to adjuvant chemotherapy for estrogen receptor-positive tumors [12, 13]. Interestingly, Oncotype DX was originally developed as a prognostic biomarker. In fact, prognostic gene expression signatures are fairly common in breast cancer [12, 14]. The reader may note that Oncotype DX was treated as a single biomaker and referred to as a gene expression based predictive classifier [3].

Statistically, predictive associations are identified using models with an interaction between a candidate biomarker and targeted therapy [15], whereas prognostic biomarkers are identified as significant main effects [16]. Thus, analysis strategies for identifying prognostic markers are often unsuitable for personalized medicine [17, 18]. In fact, the discovery of predictive biomarkers requires specific statistical techniques for data-analysis that optimize the combination of competing therapies with candidate genetic features and characteristics of the patient and disease. Recently, many statistical approaches have been developed providing researchers with new tools for identifying potential biomarkers. However, the usefulness of these recent advances has not been fully recognized by the oncology community, and the scope of their applications has not been summarized.

In this paper, we provide an overview of statistical methods for establishing optimal treatment rules for personalized medicine and discuss specific examples in various medical contexts with oncology as an emphasis. We also point the reader to statistical software when available. The various approaches enable investigators to ascertain the extent to which one should expect a new untreated patient to respond to each candidate therapy and thereby select the treatment that maximizes the expected therapeutic response for the specific patient [3, 19].  Section 2 discusses the limitations of conventional approaches based on post hoc stratified analysis.  Section 3 offers an overview of the process for the development of personalized regimes.  Section 4 discusses the selection of an appropriate statistical method for different types of clinical outcomes and data sources.  Section 5 presents technical details for deriving optimal treatment selection rules. In Section 6, we discuss approaches for evaluating model performance and assessing the extent to which treatment selection using the derived optimal rule is likely to benefit future patients.

## 2. Limitations of Subgroup Analysis

Cancer is an inherently heterogeneous disease. Yet, often efforts to personalize therapy rely on the application of analysis strategies that neglect to account for the extent of heterogeneity intrinsic to the patient and disease and therefore are too reductive for personalizing treatment in many areas of oncology [20–23]. Subgroup analysis is often used to evaluate treatment effects among stratified subsets of patients defined by one or a few baseline characteristics [23–26]. For example, Thatcher et al. [21] conducted a series of preplanned subgroup analyses for refractory advanced non-small-cell lung cancer patients treated with Gefitinib plus best supportive care against placebo. Heterogeneous treatment effects were found in subgroups defined by smoking status; that is, significant prolonged survival was observed for nonsmokers, while no treatment benefit was found for smokers.

Though very useful when well planned and properly conducted, the reliance on subgroup analysis for developing personalized treatment has been criticized [24, 25]. Obviously, a subgroup defined by a few factors is inadequate for characterizing individualized treatment regimes that depends on multivariate synthesis. Moreover, post hoc implementation of multiple subgroup analyses considers a set of statistical inferences simultaneously (multiple testing), and errors, such as incorrectly rejecting the null hypothesis, are likely to occur. The extent to which the resulting inference inflates the risk of a false positive finding can be dramatic [23]. Take, for example, a recent study that concluded that chemotherapy followed by tamoxifen promises substantial clinical benefit for postmenopausal women with ER negative, lymph node-negative breast cancer [27] through post hoc application subgroup analysis. Subsequent studies failed to reproduce this result, concluding instead that the regime's clinical effects were largely independent of ER status [28], but may depend on other factors including age.

## 3. Personalized Medicine from a Statistical Perspective

**Figure 1:**
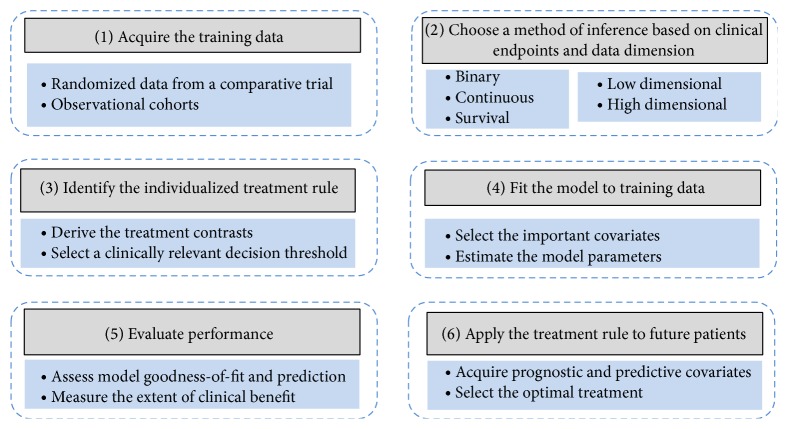
The process for using statistical inference to establish personalized treatment rules.

From a statistical perspective, personalized medicine is a process involving six fundamental steps provided in Figure 1 [20, 29, 30]. Intrinsic to any statistical inference, initially one must select an appropriate method of inference based on the available source of training data and clinical endpoints (e.g., steps (1) and (2)). Step (3) is the fundamental component of personalized treatment selection, deriving the individualized treatment rule (ITR) for the chosen method of inference. An ITR is a decision rule that identifies the optimal treatment given patient/disease characteristics [31, 32].  Section 5 is dedicated to the topic of establishing ITRs for various statistical models and types of clinical endpoints that are commonly used to evaluate treatment effectiveness in oncology.

Individualized treatment rules are functions of model parameters (usually treatment contrasts reflecting differences in treatment effects) which must be estimated from the assumed statistical model and training data. Statistical estimation takes place in step 4. The topic is quite general, and it thus is not covered in detail owning to the fact that other authors have provided several effective expositions on model building strategies in this context [29, 33]. After estimating the optimal treatment rule in step (4), the resulting estimated ITR's performance and reliability must be evaluated before the model can be used to guide treatment selection [34]. The manner in which one assesses the performance of the derived ITR depends on the appropriate clinical utility (i.e., increased response rate or prolonged survival duration). Evaluation of model goodness-of-fit and appropriate summary statistics that use the available information to measure the extent to which future patients would benefit from application of the ITR is conducted in step (5) and will be discussed in Section 6. The ITR is applied to guide treatment selection for a future patient based on his/her baseline clinical and genetic characteristics as the final step.

## 4. Selecting an Appropriate Method of Inference

The quality of a treatment rule depends on the aptness of the study design used to acquire the training data, clinical relevance of the primary endpoints, statistical analysis plans for model selection and inference, and quality of the data.* Randomized clinical trials* (RCT) remain the gold standard study design for treatment comparison, since randomization mitigates bias arising from treatment selection. Methods for deriving ITRs using data from RCTs are described in Section 5.1. Data from well conducted observational studies provide useful sources of information as well, given that the available covariates can be used to account for potential sources of confounding due to selection bias. Predominately, methods based on propensity scores are used to adjust for confounding [35, 36]. Approaches for establishing ITRs using observational studies are discussed in Section 5.2.

The predominate statistical challenge pertaining to the identification of predictive biomarkers is the high-dimensional nature of molecular derived candidate features. Classical regression models cannot be directly applied since the number of covariates, for example, genes, is much larger than number of samples. Many approaches have been proposed to analyze high-dimensional data for prognostic biomarkers.  Section 5.3 discusses several that can be applied to detect predictive biomarkers under proper modification.

In oncology, several endpoints are used to compare clinical effectiveness. However, the primary therapeutic goal is to extend survivorship or delay recurrence/progression. Thus, time-to-event endpoints are often considered to be the most representative of clinical effectiveness [37]. The approaches aforementioned were developed for ordinal or continuous outcomes and were thus not directly applicable for survival analysis. Methods for establishing ITRs from time-to-event endpoints often use Cox regression or accelerated failure time models [38, 39]. The later approach is particularly appealing in this context since the clinical benefits of prolonged survival time can be easily obtained [40, 41]. In Section 5.4, we will discuss both models.

The performance of ITRs for personalized medicine is highly dependent upon the extent to which the model assumptions are satisfied and/or the posited model is correctly specified. Specifically, performances may suffer from misspecification of main effects and/or interactions, random error distribution, violation of linear assumptions, sensitivity to outliers, and other potential sources of inadequacy [42]. Some advanced methodologies have been developed to overcome these issues [43], including semiparametric approaches that circumvent prespecification of the functional form of the relationship between biomarker and expected clinical response [32, 40]. In addition, optimal treatment rules can be defined without regression models, using classification approaches where patients are assigned to the treatment that provides the highest expected clinical benefit. Appropriate class labels can be defined by the estimated treatment difference (e.g., >0 versus ≤0), thereby enabling the use of machine learning and data mining techniques [42, 44, 45]. These will be discussed in Section 5.5.

## 5. Methods for Identifying Individualized Treatment Rules

This section provides details of analytical approaches that are appropriate identifying ITRs using a clinical data source. The very nature of treatment benefit is determined by the clinical endpoint. While extending overall survival is the ultimate therapeutic goal, often the extent of reduction in tumor size as assessed by RECIST criteria (http://www.recist.com/) is used as a categorical surrogate for long-term response. Alternatively, oncology trials often compare the extent to which the treatment delays locoregional recurrence or disease progression. Therefore, time-to-event and binary (as in absence/presence of partial or complete response) are the most commonly used endpoints in oncologic drug development [37, 46].

Let *Y* denote the observed outcome such as survival duration or response to the treatment, and let *A* ∈ {0,1} denote the treatment assignment with 0 indicating standard treatment and 1 for a new therapy. Denote the collection of observable data for a previously treated patient by (*Y*, *A*, **X**), where **X** = *X*_1_, *X*_2_,…, *X*_*p*_, represents a vector of values for the *p* biomarkers under study. Quantitatively, the optimal ITR derives from the following equation relating the observed response to the potential outcome attained under the alternative treatment

(1) $$\begin{array}{c} Y=AY^{\left(1\right)}+\left(1-A\right)Y^{\left(0\right)}, \end{array}$$

where *Y* ^(1)^ and *Y* ^(0)^ denote the potential outcomes that would be observed if the subject had been assigned to the new therapy or the standard treatment, respectively [32, 43]. Let *E*(*Y*∣*A*, **X**) = *μ*(*A*, **X**) denote the expected value of *Y* given *A* and **X**. The optimal treatment rule follows as

(2) $$\begin{array}{c} g^{\text{opt}}\left(X\right)=I\left\{\mu \left(A=1,X\right)-\mu \left(A=0,X\right)>0\right\}, \end{array}$$

where *I*(·) is the indicator function. For instance, if *I*{*μ*(1, age > 50) − *μ*(0, age > 50) > 0} = 1, then the optimal rule would assign patients who are older than 50 to the new treatment. However, *E*(*Y*∣*A*, **X**) is actually a function of parameters, *μ*(*A*, **X**; **β**), denoted by **β**. The model needs to be “fitted” to the training data to obtain estimates of **β**, which we denote by $\hat{\beta }$. Hence for a patient with observed biomarkers **X** = **x**, the estimated optimal treatment rule is

(3) g^opt(X=x,β^)  =I{μ(A=1,X=x;β^)−μ(A=0,X=x;β^)}.

The above equation pertains to steps (3) and (4) in Figure 1; that is, the parameter estimates from a fitted model are used to construct the personalized treatment rule. The remainder of this section instructs the readers how to identify ITRs for the various data types.

We classify the statistical methods presented in this section into five categories: methods based on multivariate and generalized linear regression for analysis of data acquired from RCT (Section 5.1) and observational studies (Section 5.2); methods based on penalized regression techniques for high-dimensional data (Section 5.3); methods for survival data (Section 5.4); and advanced methods based on robust estimation and machine learning techniques (Section 5.5).

### 5.1. Multiple Regression for Randomized Clinical Trial Data

Classical generalized linear models (GLM) can be used to develop ITRs in the presence of training data derived from randomized clinical study. The regression framework assumes that the outcome *Y* is a linear function of prognostic covariates, *X*_1_; putative predictive biomarkers, *X*_2_; the treatment indicator, *A*; and treatment-by-predictive interaction, *AX*_2_:

(4) μA,X=E(Y ∣ A,X)=β0+β1X1+β2X2+Aβ3+β4X2.

Let Δ(**X**) = *E*(*Y*∣*A* = 1, *X*) − *E*(*Y*∣*A* = 0, *X*) = *μ*(*A* = 1, *X*) − *μ*(*A* = 0, *X*) denote the treatment contrast. The optimal treatment rule assigns a patient to the new treatment if Δ(**X**) > 0. For binary endpoints, the logistic regression model for *μ*(*A*, **X**) = *P*(*Y* = 1∣*A*, **X**) is defined such that

(5) log⁡μ(A,X)1−μ(A,X)=ω(A,X)=β0+β1X1+β2X2+A(β3+β4X2).

The treatment contrast Δ(**X**) can be calculated using *E*(*Y*∣*A* = *a*, *X*) = *P*(*Y* = 1∣*A* = *a*, **X**) = *e* ^*ω*(*A*,**X**)^/(1 + *e* ^*ω*(*A*,**X**)^) for *a* = 0,1, respectively. Similarly, an optimal ITR assigns a patient to the new treatment if Δ(**X**) > 0. This optimal treatment rule can be alternatively defined as *g* ^opt^(**X**) = *I*{(*β*_3_ + *β*_4_ *X*_2_) > 0} without the need to calculate the treatment contrast Δ(**X**) [43, 45].

Often one might want to impose a clinically meaningful minimal threshold, Δ(**X**) > *δ*, on the magnitude of treatment benefit before assigning patients to a novel therapy [45, 47]. For example, it may be desirable to require at least a 0.1 increase in response rate before assigning a therapy for which the long-term safety profile has yet to be established. The use of a threshold value can be applied to all methods. Without loss of generality, we assume *δ* = 0 unless otherwise specified. In addition, the reader should note that the approaches for constructing an ITR described above can be easily applied to linear regression models for continuous outcomes.

This strategy was used to develop an ITR for treatment of depression [19] using data collected from a RCT of 154 patients. In this case, the continuous outcome was based on posttreatment scores from the Hamilton Rating Scale for Depression. The authors constructed a personalized advantage index using the estimated treatment contrasts Δ(**X**), derived from five predictive biomarkers. A clinically significant threshold was selected, *δ* = 3, based on the National Institute for Health and Care Excellence criterion. The authors identified that 60% of patients in the sample would obtain a clinically meaningful advantage if their therapy decision followed the proposed treatment rule. The approaches discussed in this section can be easily implemented with standard statistical software, such as the *R* (http://www.r-project.org/) using the functions* lm* and* glm* [48].

### 5.2. Methods for Observational Data

Randomization attenuates bias arising from treatment selection, thereby providing the highest quality data for comparing competing interventions. However, due to ethical or financial constraints RCTs are often infeasible, thereby necessitating an observational study. Treatment selection is often based on a patient's prognosis. In the absence of randomization, the study design fails to ensure that patients on competing arms exhibit similar clinical and prognostic characteristics, thereby inducing bias.

However, in the event that the available covariates capture the sources of bias, a well conducted observational study can also provide useful information for constructing ITRs. For example, the two-gene ratio index (HOXB13:IL17BR) was first discovered as an independent prognostic biomarker for ER+ node-negative patients using retrospective data from 60 patients [49]. These findings were confirmed on an independent data set comprising 852 tumors, which was acquired from a tumor bank at the Breast Center of Baylor College of Medicine [50]. Interestingly, the two-gene ratio index (HOXB13:IL17BR) was reported to predict the benefit of treatment with letrozole in one recent independent study [51].

Methods based on propensity scores are commonly used to attenuate selection bias [35]. In essence, these approaches use the available covariates to attempt to diminish the effects of imbalances among variables that are not of interest for treatment comparison. Moreover, they have been shown to be robust in the presence of multiple confounders and rare events [52]. Generally, after adjusting for bias using propensity scores, the same principles for deriving ITRs from RCTs may be applied to the observational cohort.

The propensity score characterizes the probability of assigning a given treatment *A* from the available covariates, **X** [35]. Using our notation, the propensity score is *π*(**X**, **ξ**) = *P*(*A* = 1∣**X**, **ξ**), which can be modeled using logistic regression

(6) $$\begin{array}{c} \log\left\{\frac{\pi \left(X\right)}{1-\pi \left(X\right)}\right\}=\xi_{0}+\xi_{1}X_{1}+\xi_{2}X_{2}+\xi_{3}X_{3}+\cdots +\xi_{p}X_{p}, \end{array}$$

where *p* is the number of independent variables used to construct the propensity score and *ξ*_*j*_ represents the *j*th regression coefficient, which characterizes the *j*th covariate's partial effect. After fitting the data to obtain estimates for the regression coefficients, $\hat{\xi }$, the estimated probability of receiving new treatment can be obtained for each patient, $\hat{\pi }(X_{i})=\pi (X_{i},\hat{\xi })$, by inverting the logit function. The event that $\hat{\xi }\approx 0$ implies that the measured independent variables are reasonably “balanced” between treatment cohorts. In practice, one often includes as many baseline covariates into the propensity score model as permitted by the sample size.

Methods that use propensity scores can be categorized into four categories: matching, stratification, adjusting, and inverse probability weighted estimation [36, 53]. Matching and stratification aim to mimic RCTs by defining a new dataset using propensity scores such that outcomes are directly comparable between treatment cohorts [53]. These two approaches are well suited for conventional subgroup analysis but their application to personalized medicine has been limited. Regression adjustment or simply adjusting can be used to reduce bias due to residual differences in observed baseline covariates between treatment groups. This method incorporates the propensity scores as an independent variable in a regression model and therefore can be used in conjunction with all regression-based methods [36]. Methods involving inverse probability weighted estimators will be discussed in Section 5.5.1 [43].

Of course, propensity scores methods may only attenuate the effects of the important confounding variables that have been acquired by the study design. Casual inference in general is not robust to the presence of unmeasured confounders that influenced treatment assignment [35, 54, 55]. For the development of ITRs, predictive and important prognostic covariates can be incorporated in the regression model for the clinical outcome *Y* along with the propensity scores, while other covariates may be utilized only in the model for estimating the propensity scores. Hence, propensity score methods may offer the researcher a useful tool for controlling for potential confounding due to selection bias and maintaining a manageable number of prognostic and predictive covariates.

### 5.3. Methods for High-Dimensional Biomarkers

The methods presented in the previous sections are appropriate for identifying an ITR using a small set of biomarkers (low-dimensional). However, recent advances in molecular biology in oncology have enabled researchers to acquire vast amounts of genetic and genomic characteristics on individual patients. Often the number of acquired genomic covariates will exceed the sample size. Proper analysis of these high-dimensional data sources poses many analytical challenges. Several methods have been proposed specifically for analysis of high- dimensional covariates [56], although the majority of these methods are well suited only for the analysis of prognostic biomarkers. In what follows, we introduce variable selection methods that were developed to detect predictive biomarkers from high-dimensional sources as well as describing how to construct optimal ITRs from the final set of biomarkers.

An appropriate regression model can be defined generally as $E(Y|A,X)=h_{0}(X)+A(\tilde{X}\beta )$, where *h*_0_(**X**) is an unspecified baseline mean function, **β** = (*β*_0_, *β*_1_,…, *β*_*q*_)^*T*^ is a column vector of regression coefficients, and $\tilde{X}=(1,X)$ the design matrix. Subscript *q* denotes the total number of biomarkers, which may be larger than the sample size *n*. An ITR derives from evaluating the interactions in $A(\tilde{X}\beta )$, not the baseline effect of the high-dimensional covariates *h*_0_(**X**) [32]. Technically, function $A(\tilde{X}\beta )=A(\beta_{0}+\beta_{1}X_{1}+\beta_{2}X_{2}+\cdots +\beta_{q}X_{q})$ cannot be uniquely estimated using traditional maximum likelihood-based methods when *q* > *n* [57]. Yet, practically, many of the available biomarkers may not influence the optimal ITR [31]. Thus, the process for identify ITRs from a high-dimensional source requires that we first identify a sparse subset of predictive biomarkers that can be utilized for constructing the ITR.

Parameters for the specified model can be estimated using the following loss function:

(7) $$\begin{array}{c} L_{n,\phi }\left(\beta ,\gamma \right)=\frac{1}{n}\overset{n}{\underset{i=1}{\sum }}{\left[Y_{i}-\phi \left(X_{i};\gamma \right)-\tilde{X}\beta \left\{A_{i}-\pi \left(X_{i}\right)\right\}\right]}^{2}, \end{array}$$

where *ϕ*(**X**; **γ**) represents any arbitrary function characterizing the “baseline” relationship between **X** and **Y** (e.g., an intercept or an additive model). Here we let *π*(**X**_*i*_) = *P*(*A*_*i*_ = 1∣**X**_*i*_) denote either a propensity score (for observational data) or a randomization probability (e.g., 0.5 given 1 : 1 randomization) for RCT data. If *π*(**X**) is known, estimation using this model yields unbiased estimates (asymptotically consistent) of the interaction effects **β** even if the main effects are not correctly specified, providing a robustness [32].

Penalized estimation provides the subset of relevant predictive markers that are extracted from the nonzero coefficients of the corresponding treatment-biomarker interaction terms of

(8) $$\begin{array}{c} \hat{\beta }=\arg\underset{\beta }{\min}\left\{L_{n,\phi }\left(\beta ,\gamma \right)+\lambda_{n}\overset{p+1}{\underset{j=1}{\sum }}J\left|\beta_{j}\right|\right\}, \end{array}$$

where *λ*_*n*_ is a tuning parameter which is often selected via cross validation and *J* is a shrinkage penalty. Different choices of *J* lead to different types of estimators. For example, the lasso penalized regression corresponds to *J* = 1 [58] and the adaptive lasso to $J=\omega_{j}=1/|{\hat{\beta }}_{\text{init},j}|$, where ${\hat{\beta }}_{\text{init},j}$ is an initial estimate of *β*_*j*_ [59]. With little modification, 8 can be solved using the LARS algorithm implemented with the *R* package of *lars* [32, 60, 61]. As we have shown before, a treatment rule can be defined from the parameter estimates as *I*{*β*_0_ + *β*_1_ *X*_1_ + *β*_2_ *X*_2_ + ⋯+*β*_*q*_ *X*_*q*_ > 0}.* Note* this generic form may have zero estimates for some coefficients (e.g., ${\hat{\beta }}_{2}={\hat{\beta }}_{5}=\cdots ={\hat{\beta }}_{q}=0$); hence an ITR can be equivalently constructed from the final estimated nonzero coefficients and the corresponding covariates.

Alternative penalized regression approaches include SCAD [62] and elastic-net [63]. All penalized approaches produce sparse solutions (i.e., identifying a small subset of predictive biomarkers); however the adaptive lasso is less effective when *p* > *n*. Methods that produce nonsparse models, such as ridge regression [57], are less preferable since ITRs based on many biomarkers are often unstable and less useful in practice [31]. Several packages in *R* offer implementation of penalized regression, such as *parcor* for ridge, lasso and adaptive lasso, and *ncvreg* for SCAD [64, 65].

Lu et al. [32] used a penalized regression approach to analyze data from the AIDS Clinical Trials Group Protocol 175 (ACTG175) [66]. In this protocol, 2,000 patients were equally randomized to one of four treatments: zidovudine (ZDV) monotherapy, ZDV + didanosine (ddI), ZDV + zalcitabine, and ddI monotherapy. CD4 count at 15–25 weeks postbaseline was the primary outcome and 12 baseline covariates were included in the analysis. The resulting treatment rule favored the combined regimes over ZDV monotherapy. Moreover, the treatment rule determined that ZDV + ddI should be preferred to ddI when *I*(71.59 + 1.07 × age − 0.18 × CD40 − 33.57 × homo) = 1, where CD40 represents baseline CD4 counts and homo represents homosexual activity. Based on this treatment rule, 878 patients would have benefited from treatment with ZDV + ddI.

### 5.4. Survival Analysis

Heretofore, we have discussed methods for continuous or binary outcomes, yet often investigators want to discern the extent to which a therapeutic intervention may alter the amount of time required before an event occurs. This type of statistical inference is referred to broadly as survival analysis. One challenge for survival analysis is that the outcomes may be only partially observable at the time of analysis due to censoring or incomplete follow-up. Survival analysis has been widely applied in cancer studies, often in association studies aimed to identify prognostic biomarkers [56, 67]. Here we discuss two widely used models for deriving ITRs using time-to-event data, namely, Cox regression and accelerated failure time models.

The Cox regression model follows as

(9) $$\begin{array}{c} \lambda \left(t\;|\;X,A\right)=\lambda_{0}\left(t\right)\exp\left\{\beta_{1}X_{1}+\beta_{2}X_{2}+A\left(\beta_{4}+\beta_{5}X_{2}\right)\right\}, \end{array}$$

where *t* is the survival time, *λ*_0_(*t*) is an arbitrary baseline hazard function, and *X*_1_, *X*_2_ represent prognostic and predictive biomarkers, respectively. Each *β* characterizes the multiplicative effect on the hazard associated with a unit increase in the corresponding covariate. Therefore, Cox models are referred to as proportional hazards (PH) models.

Several authors have provided model building strategies [29] and approaches for treatment selection [20, 30, 68]. Following the previously outlined strategy, a naive approach for deriving an ITR uses the hazard ratio (new treatment versus the standard) as the treatment contrast, which can be calculated as Δ(**X**) = exp⁡(*β*_4_ + *β*_5_ *X*_2_). The ITR therefore is *I*{(*β*_4_ + *β*_5_ *X*_2_) < 0}. There are obvious limitations to this approach. First, violations of the PH assumption yield substantially misleading results [69]. Moreover, even when the PH assumption is satisfied, because the Cox model does not postulate a direct relationship between the covariate (treatment) and the survival time, the hazard ratio fails to measure the extent to which the treatment is clinically valuable [38, 70].

Accelerated failure time (AFT) models provide an alternative semiparametric model. Here we introduce its application for high-dimensional data. Let *T* and *C* denote the survival and censoring times, and denote the observed data by $\left(\tilde{T},\delta ,A,X\right)$ where $\tilde{T}=\min(T,C)$ and *δ* = *I*(*T* < *C*). Define the log survival time as *Y* = log⁡(*T*); a semiparametric regression model is given as $E(Y|A,X)=h_{0}(X)+A(\tilde{X}\beta )$, where *h*_0_(**X**) is the unspecified baseline mean function. Similar to the previous section, the treatment rule is *I*{(*β*_0_ + *β*_1_ *X*_1_ + *β*_2_ *X*_2_ + ⋯+*β*_*q*_ *X*_*q*_) > 0}. Under the assumption of independent censoring, the AFT model parameters can be estimated by minimizing the following loss function:

(10) $$\begin{array}{c} L_{n,\phi }\left(\beta \right)=\frac{1}{n}\overset{n}{\underset{i=1}{\sum }}\frac{\delta_{i}}{\hat{G}\left({\tilde{T}}_{i}\right)}{\left[{\tilde{Y}}_{i}-\phi \left(X_{i};\gamma \right)-\tilde{X}\beta \left\{A_{i}-\pi \left(X_{i}\right)\right\}\right]}^{2}, \end{array}$$

where ${\tilde{Y}}_{i}=\log({\tilde{T}}_{i})$, *π*(**X**_*i*_) = *P*(*A*_*i*_ = 1∣**X**_*i*_) is the propensity score or randomization probability, $\hat{G}(\cdot )$ is the Kaplan-Meier estimator of the survival function of the censoring time, and *ϕ*(**X**; **γ**) characterizes any arbitrary function.

This method can be extended to accommodate more than two treatments simultaneously by specifying appropriate treatment indicators. For instance, the mean function can be modeled as $E(Y|A,X)=h_{0}(X)+A_{\left(1\right)}\{\tilde{X}\beta_{\left(1\right)}\}+A_{\left(2\right)}\{\tilde{X}\beta_{\left(2\right)}\}$ for two treatment drugs versus the standard care. The ITR assigns the winning drug. Note this work was proposed by [40] and is an extension of [32] to the survival setting. Hence, it shares the robustness property and can be applied to observational data. For implementation, the same procedure can be followed to obtain estimates, with one addition step of calculating $\hat{G}({\tilde{T}}_{i})$. There are several *R* packages for Kaplan-Meier estimates and Cox regression models. These sources can be found at http://cran.r-project.org/web/views/Survival.html. More details pertaining to statistical methods for survival analysis can be found here [71]. To compare treatment rules constructed from Cox and AFT models, for example, methods for measuring the extent of clinical effectiveness for an ITR will be discussed in Section 6.

We here present an example when an AFT model was used to construct an ITR for treatment of HIV [40]. The example derives from the AIDS Clinical Trials Group Protocol 175 that was discussed in Section 5.3 [32, 66]. In this case, the primary outcome variable was time (in days) to first ≥50% decline in CD4 count or an AIDS-defining event or death. A total of 12 covariates and four treatments (ZDV, ZDV + ddI, ZDV + zalcitabine, and ddI) were included. The four treatments were evaluated simultaneously. Patients receiving the standard care of ZDV monotherapy were considered as the reference group. Hence, three treatment contrasts (*I*_ZDV+ddI_, *I*_ZDV+zalcitabine_, and *I*_ddI_) were combined with various putative predictive covariates and compared with ZDV monotherapy. For example, gender was detected as the predictive covariate only for ddI monotherapy. The investigators assumed *ϕ*(**X**; **γ**) = *γ*_0_. The treatment rule recommended 1 patient for ZDV monotherapy, while 729, 1216, and 193 patients were recommended for ZDV + ddI, ZDV + zalcitabine, and ddI, respectively.

### 5.5. Advanced Methods

#### 5.5.1. Robust Inference

The performances of ITRs heretofore presented depend heavily on whether the statistical models were correctly specified. Recently there has been much attention focused on the development of more advanced methods and modeling strategies that are robust to various aspects of potential misspecification. We have already presented a few robust models that avoid specification of functional parametric relationships for main effects [32, 40]. Here, we introduce two more advanced methods widely utilized for ITRs that are robust to the type of misspecification issues commonly encountered in practice [42, 43].

Recall that the ITR for a linear model *E*(*Y*∣*A* = *a*, **X**) = *μ*(*A* = *a*, **X**; **β**) with two predictive markers follows as *g*(**X**, **β**) = *I*{(*β*_4_ + *β*_5_ *X*_2_ + *β*_6_ *X*_3_) > 0}, where *a* = 0,1. The treatment rule of *g*(**X**, **β**) may use only a subset of the high-dimensional covariates (e.g., {*X*_2_, *X*_3_}), but it always depends on the correct specification of *E*(*Y*∣*A* = *a*, **X**). Defining a scaled version of *β* as *η*(*β*), the corresponding ITR is *g*(**η**, **X**) = *g*(**X**, **β**) = *I*(*X*_3_ > *η*_0_ + *η*_1_ *X*_2_), where *η*_0_ = −*β*_4_/*β*_6_ and *η*_1_ = *β*_5_/*β*_6_. If the model for *μ*(*A*, **X**; **β**) is indeed correctly specified, the treatment rules of *g*(**X**, **β**) and *g*(**η**, **X**) lead to the same optimal ITR. Hence, the treatment rule parameterized by **η** can be derived from a regression model or may be based on some key clinical considerations which enable evaluation of *g*(**η**, **X**) directly without reference to the regression model for *μ*(*A*, **X**; **β**).

Let *C*_*η*_ = *Ag*(**η**, **X**)+(1 − *A*){1 − *g*(**η**, **X**)}, where *C*_*η*_ = 1 indicates random assignment to an intervention that is recommended by the personalized treatment rule *g*(**η**, **X**). Let $\pi (X;\hat{\gamma })$ denote the randomization ratio or the estimated propensity score (as in previous section), and $m(X;\eta ,\hat{\beta })$ denote the potential outcome under the treatment rule estimated from the following model *E*(*Y*∣*A* = *a*, **X**) = *μ*(*A*, **X**; **β**). For example, if the treatment rule *g*(**η**, **X**) = 1, then $m(X;\eta ,\hat{\beta })=g(\eta ,X)\mu (A=1,X;\hat{\beta })+\{1-g(\eta ,X)\}\mu (A=0,X;\hat{\beta })=\mu (A=1,X;\hat{\beta })$. Two estimators of the expected response to treatment, the inverse probability weighted estimator (IPWE) and doubly robust AIPWE, are given as follows:

(11) IPWEη=1n∑i=1nCη·iYiπcXi;η,γ^=1n∑i=1nCη·iYiπXi;γ^Ai1−πXi;γ^1−Ai,AIPWEη=1n∑i=1nCη·iYiπc(Xi;η,γ^)llllllllllllll−Cη·iYi−πc(Xi;η,γ^)πc(Xi;η,γ^)m(Xi;η,β^),

where $\pi_{c}(X_{i};\eta ,\hat{\gamma })=\pi (X;\hat{\gamma })g(\eta ,X)+\{1-\pi (X;\hat{\gamma })\}\{1-g(\eta ,X)\}$. The optimal treatment rule follows as $\hat{g}(\hat{\eta },X=x)$, where $\hat{\eta }$ is estimated from the above models; a constraint, such as ‖**η**‖ = 1, is imposed to obtain a unique solution $\hat{\eta }$ [43]. If the propensity score is correctly specified, the IPWE estimator yields robust (consistent) estimates; AIPWE is considered a doubly robust estimator since it produces consistent estimates when either propensity score or the model *E*(*Y*∣*A* = *a*, **X**) is misspecified, but not both [42, 43]. The companion *R* code is publicly available at http://onlinelibrary.wiley.com/doi/10.1111/biom.12191/suppinfo.

#### 5.5.2. Data Mining and Machine Learning

The methods presented in Section 5.5.1 are robust against misspecification of regression models. Yet, they often require prespecification of the parametric form for the treatment rule (e.g., *I*(*X*_3_ > *η*_0_ + *η*_1_ *X*_2_)), which can be practically challenging [44]. Well established classification methods and other popular machine learning techniques can alternatively be customized to define treatment selection rules [44, 72, 73]; these methods avoid prespecification of the parametric form of the ITR. An ITR can be defined following a two-step approach: in the first step, treatment contrasts are estimated from a posited model and in the second step classification techniques are applied to determine the personalized treatment rules. For example, when only two treatments are considered, a new variable *Z* can be defined based on the treatment contrast; that is, *Z* = 1 if Δ(**X**) = {*μ*(*A* = 1, **X**) − *μ*(*A* = 0, **X**)} > 0 and *Z* = 0 otherwise. The absolute value of the treatment contrast *W*_*i*_ = |Δ(**X**)| can be used in conjunction with a classification technique to define an appropriate ITR [44].

Unlike classification problems wherein the class labels are observed for the training data, the binary “response” variable *Z*, which serves as the class label, is not available in practice. Specifically, patients who are in the class *Z* = 1 have {*μ*(*A* = 1, **X**) > *μ*(*A* = 0, **X**)} and should therefore be treated with the new therapy; however these quantities need to be estimated, since patients are typically assigned to only one of the available treatments. This imparts flexibility for estimation of the optimal treatment regimes, since any of the previously discussed regression models and even some ensemble prediction methods such as random forest [74] can be used to construct the class labels ${\hat{Z}}_{i}$ and weights ${\hat{W}}_{i}$ [44]. An ITR can be estimated from the dataset $\left\{{\hat{Z}}_{i},X_{i},{\hat{W}}_{i}\right\}$ using any classification approach, where ${\hat{W}}_{i}$ are subject specific misclassification weights [44, 45]. This includes popular classification methods such as adaptive boosting [75], support vector machines [76], and classification and regression trees (CART) [77]. At least one study has suggested that SVM outperforms other classification methods in this context, whereas random forest and boosting perform comparatively better than CART [78]. However, the performances of these classification algorithms are data dependent. Definitive conclusion pertaining to their comparative effectiveness in general has yet to be determined [78]. It shall be also noted that these classification methods can be also applied to high-dimensional data [45, 72].

One special case of this framework is the “virtue twins” approach [45]. Specifically, in the first step a random forest approach [74] is used to obtain the treatment contrasts. Then in the second step CART is used to classify subjects to the optimal treatment regime. The approach can be easily implemented in *R* using packages of* randomForest* [79] and* rpart* [80]. Very recently, Kang et al. [42] proposed a modified version of the adaptive boosting technique of Friedman et al. [75]. The algorithm iteratively fits a simple logistic regression model (“working model”) to estimate *P*(*Y* = 1∣*A*, **X**) and at each stage assigns higher weights to subjects whose treatment contrast is near zero. After a prespecified stopping criterion is met, an average of the treatment contrasts $\bar{\Delta }(X)$ is calculated for each patient using all models fitted at each iteration. A subject is assigned to the new therapy if $\bar{\Delta }(X)>0$. The *R* code for the aforementioned boosting methods is publicly available at http://onlinelibrary.wiley.com/doi/10.1111/biom.12191/suppinfo.

Lastly, we present a breast cancer example where several biomarkers were combined to construct an optimal ITR. The data was collected in the Southwest Oncology Group (SOWG)-SS8814 trial [13] and analyzed with the machine learning approach of Kang et al. [42]. Three hundred and sixty-seven node-positive, ER-positive breast cancer patients were selected from the randomized trial of SOWG. A total of 219 received tamoxifen plus adjuvant chemotherapy and 148 was given tamoxifen alone. The outcome variable was defined as breast cancer recurrence at 5 years. The authors selected three genes, which had presented treatment-biomarker interactions in a multivariate linear logistic regression model [42]. Data were analyzed with logistic models, IPWE, AIPWE, logistic boosting, a single classification tree with treatment-biomarker interactions, and the proposed boosting approach with a classification tree as the working model. Each method identified different patient cohorts that could benefit from tamoxifen alone: these cohorts consisted of 184, 183, 128, 86, 263, and 217 patients, respectively (see Table  5 in [42]). In this analysis, the clinical benefits provided by these 6 treatment rules were not statistically different. Hence, investigators need to evaluate and compare ITRs in terms of the extent of expected clinical impact. This is considered in the next section.

## 6. Performance Evaluation for Individualized Treatment Rules

Heretofore, we have discussed various methodologies for the construction of ITR, while their performances need to be assessed before these rules can be implemented in clinical practice. Several aspects pertaining to the performance of a constructed ITR need to be considered. The first one is how well the ITR fits the data, and the second is how well the ITR performs compared with existing treatment allocation rules. The former is related to the concept of goodness-of-fit or predictive performance [34]. As the true optimal treatment groups are hidden, model fits may be evaluated by measuring the congruity between observed treatment contrasts and predicted ones [34, 47]. More details can be found in a recent paper by Janes et al. [47]. Performances of ITRs can be compared via assessment of a global summary measure, for example, prolonged survival time or reduced disease rate [40, 42]. Summary measures are also very useful for evaluating the extent to which an ITR may benefit patients when applied in practice. Moreover, it is essential that performance of an ITR is considered in comparison to business-as-usual procedures such as a naive rule that randomly allocates patients to treatment [81]. Summary measures will be discussed in Section 6.1. The effectiveness of an ITR should go beyond the training data set used to construct a treatment rule; cross-validation and bootstrapping techniques are often employed to assess the impact of ITRs on future patients [81] and will be discussed in Section 6.2.

### 6.1. Summary Measures

ITRs may be derived from different methodologies, and comparisons should be conducted with respect to the appropriate clinically summaries. A few summary measures for different types of outcomes have been proposed [19, 40, 42]; these measures quantify the direct clinical improvements obtained by applying an ITR in comparison with default methods for treatment allocation.

*Binary Outcomes*. Clinical effectiveness for binary clinical response is represented by the difference in disease rates (or treatment failure) induced by ITR versus a default strategy that allocates all patients to a standard treatment [42, 47, 82]. Let *g* ^opt^(**X**) = *I*{*μ*(*A* = 1, **X**) − *μ*(*A* = 0, **X**) < 0}, be an optimal ITR. This difference is formally defined as

(12) ΘBgoptX =P(Y=1 ∣ A=0)  −∑a=01PY=1 ∣ A=a,goptX=aPgoptX=a =PY=1 ∣ A=0,gopt(X)=1lllllllll−PY=1 ∣ A=1,gopt(X)=1PgoptX=1.

Note *μ*(*A*, **X**) needs to be estimated to construct the ITR yet parameters **β** are omitted for simplicity. Larger values of Θ_*B*_{*g* ^opt^(**X**)} indicate increased clinical value for the biomarker driven ITR. A subset of patients that are recommended for new treatment (*A* = 1) under an ITR may have been randomly selected to receive it, while the remaining subset of “unlucky” patients would have received the standard treatment [19]. The summary measure of Θ_*B*_{*g* ^opt^(**X**)} characterizes a weighted difference in the disease rates between the standard and the new treatments in a population wherein the constructed optimal ITR would recommend the new treatment *g* ^opt^(**X** = 1). The weight is the proportion of patients identified by the optimal ITR for the new treatment and can be empirically estimated using the corresponding counts. For example, *P*{*g* ^opt^(**X**) = 1} can be estimated using the number of patients recommended for the new treatment divided by the total sample size. A similar summary statistic can be derived for an alternative strategy allocating all patients to the new treatment. The summary could be applied to the aforementioned breast cancer example [42], for example, with the aim of finding a subgroup of patients who were likely to benefit from adjuvant chemotherapy, while those unlikely to benefit would be assigned tamoxifen alone to avoid the unnecessary toxicity and inconvenience of chemotherapy.

*Continuous Variables*. Another strategy for continuous data compares outcomes observed for “lucky” subjects, those who received the therapy that would have been recommended by the ITR based [81]. Further, one business-as-usual drug allocation procedure is randomizing treatment and standard care at the same probability of 0.5. A summary statistic is to measure the mean outcome under ITR compared to that obtained under random assignment, for instance, the mean decrease in Hamilton Rating Scale for Depression as discussed in Section 5.1 [19]. Define the summary measure as Θ_*C*_{*g* ^opt^(**X**)} = *μ*{*g* ^opt^(**X**), **X**} − *μ*{*g* ^rand^(**X**), **X**}, where *g* ^rand^(**X**) represents the randomization allocation procedure. The quantity of *μ*{*g* ^*opt*^(**X**), **X**} represents the mean outcome under the constructed IRT that can be empirically estimated from the “lucky” subjects, and *μ*{*g* ^rand^(**X**), **X**} can be estimated empirically from the sample means.

Alternatively, an ITR may be compared to an “optimal” drug that has showed universal benefits (a better drug on average) in a controlled trial. The clinical benefits of an “optimal” drug can be defined as *μ*{*g* ^best^(**X**), **X**} = max⁡{*μ*(*A* = 0, **X**), *μ*(*A* = 1, **X**)}; *μ*(*A* = *a*, **X**), and can be empirically estimated from the sample means of the new and standard treatments, respectively. Then the alternative summary measure is defined as Θ_*C*alt_ = {*g* ^opt^(**X**)} = *μ*{*g* ^opt^(**X**), **X**} − *μ*{*g* ^best^(**X**), **X**}.

*Survival Data*. For survival data, a clinically relevant measure is mean overall (or progression free) survival time. As survival time is continuous in nature, the identical strategy provided above for continuous outcomes can be employed here. However, because the mean survival time may not be well estimated from the observed data due to a high percentage of censored observations [40], an alternative mean restricted survival duration was proposed and defined as the population average event-free durations for a restricted time of *t* ^*^ [41, 83]. Often *t* ^*^ is chosen to cover the trial's follow-up period. Mathematically, it can be calculated by integrating the survival function of *S*(*t*) over the domain of (0, *t* ^*^), that is, *μ*{*g* ^opt^(**X**), **X**, *t* ^*^} = ∫_0_^t^*^^ *S*(*t*)*dt*, and often estimated by the area under the Kaplan-Meier curve up to *t* ^*^ [84]. Thus, an ITR's potential to prolong survival can be calculated as Θ_*S*_{*g* ^opt^(**X**), *t* ^*^} = *μ*{*g* ^opt^(**X**), **X**, *t* ^*^} − *μ*{*g* ^rand^(**X**), **X**, *t* ^*^}.

### 6.2. Assessing Model Performance

The summaries heretofore discussed evaluate an optimal ITR for a given model and estimating procedure. Because these quantities are estimated conditionally given the observed covariates, they neglect to quantify the extent of marginal uncertainty for future patients. Hence an ITR needs to be internally validated if external data is not available [34]. Cross-validation (CV) and bootstrap resampling techniques are commonly used for this purpose [19, 42, 45, 81], and expositions on both approaches are well described elsewhere [33, 85, 86].

We here briefly introduce a process that was proposed by Kapelner et al. [81] in the setting of personalized medicine. Tenfold CV is commonly used in practice, where the whole data is randomly partitioned into 10 roughly equal-sized exclusive subsamples. All methods under consideration are applied to 9/10 of the data, excluding 1/10 as an independent testing data set. The process is repeated 10 times for each subsample. Considering the assignments recommended by the optimal ITRs, the summary measures can be calculated using results from each testing fold [45]. The CV process gives the estimated summary measures, and its variation can be evaluated using bootstrap procedures. Specifically, one draws a sample with replacement from the entire data and calculates the summary measure from 10-fold CV. This process will be repeated *B* times, where *B* is chosen for resolution of the resulting confidence intervals [81]. Using the summary measures as *B* new random samples, the corresponding mean and variances can be calculated empirically. Note that the summary measures compare two treatment rules, one for the optimal ITR and another naive rule (e.g., randomization).

The above procedure can be applied to all the methods we have discussed so far. The *R* software package *TreatmentSelection* (http://labs.fhcrc.org/janes/index.html) can be used to implement these methods for evaluating and comparing biomarkers for binary outcomes [47]. Very recently, an inferential procedure was proposed for continuous outcomes that is implemented in the publicly available *R* package “Personalized Treatment Evaluator” [81, 87]. Both methods consider data from RCTs with two arms for comparative treatments. These methods are, in general, applicable to regression model based methods but are not suitable for approaches based on classification techniques or penalized regression.

Next we present two examples. Recall in Section 5.5 that Kang et al. [42] reported the estimated clinical benefits of an ITR for breast cancer when compared to the default strategy of assigning all patients to adjuvant chemotherapy. The proposed approach (based on boosting and classification trees) achieved the highest value of the summary measure at 0.081 with 95% confidence interval (CI) (0.000,0.159) [42]. In the second example, introduced in Section 5.1 [19], the authors calculated the mean score of the Hamilton Rating Scale for Depression for two groups of subjects; groups were defined by randomly assigning patients to the “optimal” and “nonoptimal” therapy as defined by the ITR. The reported difference between the two groups was −1.78 with a *P* value of 0.09, which fails to attain a clinical significant difference of 3 [19]. The same data was analyzed by Kapelner et al. [81]. Following the discussed procedure, the authors reported the estimated values (and 95% CI) of Θ_*C*_{*g* ^opt^(**X**)} and Θ_*C*alt_{*g* ^opt^(**X**)} as −0.842(−2.657, −0.441) and −0.765(−2.362,0.134), respectively. The results, which fail to achieve clinical significance, were based on rigorous statistical methods and thus can be considered reliable estimates of the ITR's performance.

## 7. Discussion

As our understanding tumor heterogeneity evolves, personalized medicine will become standard medical practice in oncology. Therefore, it is essential that the oncology community uses appropriate analytical methods for identifying and evaluating the performance of personalized treatment rules. This paper provided an exposition of the process for using statistical inference to establish optimal individualized treatment rules using data acquired from clinical study. The quality of an ITR depends on the quality of the design used to acquire the data. Moreover, an ITR must be properly validated before it is integrated into clinical practice. Personalized medicine in some areas of oncology may be limited by the fact that biomarkers arising from a small panel of genes may never adequately characterize the extent of tumor heterogeneity inherent to the disease. Consequently, the available statistical methodology needs to evolve in order to optimally exploit global gene signatures for personalized medicine.

The bulk of our review focused on statistical approaches for treatment selection at a single time point. The reader should note that another important area of research considers optimal dynamic treatment regimes (DTRs) [88, 89], wherein treatment decisions are considered sequentially over the course of multiple periods of intervention using each patient's prior treatment history. Zhao and Zeng provide a summary of recent developments in this area [90].
